# Manipulative Surgery

**Published:** 1917-04-21

**Authors:** Montagu Lomax


### THE EDITOR'S LETTER-BOX.

### Manipulative Surgery.

*To the Editor of* The Hospital.

Sir,—Your editorial footnote to the correspondence on Manipulative Surgery in the issue of The Hospital for April 7 is disingenuous in the extreme. You do not seem to be aware that you have executed a complete *volte-face*. In your leading article of March 17 you insinuated that, though Mr. Barker might be "sometimes successful," yet, as the world never heard of his failures, it was probable that the latter greatly predominated. You even suggested that Mr. Barker "rushed in where surgeons feared to tread," and likened his chances of success to those of a man who, "ignorant of the mechanism of a watch," attempted to repair it "by stirring up its mechanism with a skewer." In other words, you sneeringly insinuated that Mr. Barker was dishonest, reckless, and superlatively ignorant, and that he was only successful by accident. Yet you are disingenuous enough in The Hospital of April 7 to say: "We never denied and never doubted that Mr. Barker has had successes, and striking successes." You also say: "It appears *prima facie* that Mr. Barker does possess special skill in reducing dislocation of the semilunar cartilages of the knee, and that he has had successes in other injuries of joints." As a medical man myself, who holds no brief for Mr. Barker, but simply has an Englishman's love of fair play, will you allow me to say that disingenuousness such as this, to call it by no harsher name, is nauseating? Can you wonder that not only Mr. Barker and his friends, but all honest men, are disgusted with the manner in which he is treated by the medical press of this country? Could stronger evidence be supplied that the champions of medical orthodoxy are so mortally afraid of acknowledging Mr. Barker's possession of skill to which they can lay no claim that they are prepared to stick at no means, however disingenuous and discreditable, of minimising it to the best of their ability?

Your further contention that, should a committee of surgeons report favourably on Mr. Barker's methods, a grave risk would be run of Mr. Barker's making use of such a report "for purposes of advertisement" is näive in the extreme. The cat is let out of the bag with a vengeance. Nothing could show more plainly what it is that you really *fear*—viz., that Mr. Barker's claims should be vindicated and the methods of his traducers be exposed. That would be advertisement enough to last Mr. Barker for a lifetime, and for this reason alone you are wise to oppose it. It is a sorry business.—Yours, etc.,

Montagu Lomax, M.R.C.S., L.R.C.P.

[*** In the editorial footnote our correspondent refers to we hinted pretty plainly that the discretion of some of Mr. Barker's friends could not be relied on. The letter we now publish confirms this view. We have not room for further correspondence.—Ed. The Hospital.]

### The Royal Hospital for Incurables, Putney.

*To the Editor of* The Hospital.

